# Effect of Chitosan as Active Bio-colloidal Constituent on the Diffusion of Dyes in Agarose Hydrogel

**DOI:** 10.3390/gels9050395

**Published:** 2023-05-09

**Authors:** Martina Klučáková

**Affiliations:** Faculty of Chemistry, Brno University of Technology, Purkyňova 464/118, 612 00 Brno, Czech Republic; klucakova@fch.vutbr.cz

**Keywords:** chitosan, agarose, dyes, diffusion, sorption

## Abstract

Agarose hydrogel was enriched by chitosan as an active substance for the interactions with dyes. Direct blue 1, Sirius red F3B, and Reactive blue 49 were chosen as representative dyes for the study of the effect of their interaction with chitosan on their diffusion in hydrogel. Effective diffusion coefficients were determined and compared with the value obtained for pure agarose hydrogel. Simultaneously, sorption experiments were realized. The sorption ability of enriched hydrogel was several times higher in comparison with pure agarose hydrogel. Determined diffusion coefficients decreased with the addition of chitosan. Their values included the effects of hydrogel pore structure and interactions between chitosan and dyes. Diffusion experiments were realized at pH 3, 7, and 11. The effect of pH on the diffusivity of dyes in pure agarose hydrogel was negligible. Effective diffusion coefficients obtained for hydrogels enriched by chitosan increased gradually with increasing pH value. Electrostatic interactions between amino group of chitosan and sulfonic group of dyes resulted in the formation of zones with a sharp boundary between coloured and transparent hydrogel (mainly at lower pH values). A concentration jump was observed at a given distance from the interface between hydrogel and the donor dye solution.

## 1. Introduction

Chitosan is a crystalline polysaccharide obtained by the deacetylation of chitin, a by-product of the seafood industry [1,2]. As a result of the unique chemical structure, chitosan and its derivatives have been paid close and extensive attention as a potential bio-functional material [3] and they have prospective applications in many fields such as biomedicine, wastewater treatment, functional membranes, and flocculation [4]. Most of the commercial or practical applications of chitosan are confined to its unmodified forms [5]. However, synthesis of modified chitosan via N-substitution, O-substitution, free radical graft copolymerization, and other modification methods are developed to improve the application potential of this material [4,5,6,7,8]. Chitosan belongs to polyelectrolytes which can be found anywhere around us. In the form of charged biopolymers, such as nucleic acids and some polysaccharides and proteins, they form vital structural and functional constituents of living organisms. Additionally, they represent the crucial component of many non-living parts of nature, such as soils, waters, and sediments, where they—in the form of humus—regulate environmental and biological uptake and transport of essential nutrients as well as harmful pollutants. Similarly, chitosan can be used as an active substance able to interact with many pollutants, immobilize them, and affect their migration [9]. Therefore, it was chosen for this study as a representant of bio-polyelectrolytes able to interact with different constituents and affect their migration ability. It can be applied in natural systems as well as in artificial hydrogels. Agarose (a linear polysaccharide of red algae, made up of the repeating monomeric unit of agarobiose) is proposed as material-of-choice for the preparation of the hydrogel which can be enriched by an active substance for the investigation of the interactions during the transport [10,11]. The network of agarose chains can be interpenetrated by chitosan at higher temperatures where both compounds are dissolved, and the mixture is then easily gelled by cooling to normal temperature. The mechanical and textural properties of agarose hydrogels as well as the gelation mechanism are well understood [12,13,14]. The diffusion in agarose hydrogels has already been subject to vast concern [15,16,17,18,19,20]. Golmohamadi et al. [15] studied self- and mutual diffusion of Cd^2+^ and charged rhodamine derivatives. Lead et al. [16] determined diffusion coefficients of humic acids in agarose hydrogel and in water. They obtained values between 0.9 and 2.5 × 10^−10^ m^2^ s^−1^ which were generally 10–20% lower than in water. Gutenwik et al. [17] measured the effective diffusion coefficients of lysozyme and bovine serum albumin. They demonstrated the influence of pH and ionic strength on their diffusive properties. The same proteins were studied by Liang et al. [18]. At the considered range of agarose concentration (0.5–3.0 wt.%), the diffusion coefficients range from 4.98 to 8.21 × 10^−11^ m^2^/s for BSA and 1.15 to 1.56 × 10^−10^ m^2^/s for lysozyme, respectively. Tan et. Al. [19] applied a real-time electronic speckle pattern interferometry method to study the diffusion behavior of levofloxacin mesylate. Their results confirmed that the diffusivity of solute decreased with the increase of concentration of agarose. Its value extrapolated to infinite dilution was equal to 5.3 × 10^−10^ m^2^/s. Labille et al. [20] used fluorescence correlation spectroscopy to study the diffusion of nanometric solutes in agarose hydrogel and determined values of diffusion coefficients between 0.5 and 2.8 × 10^−10^ m^2^ s^−1^. Their results showed that, at the liquid/gel interface, a thin hydrogel layer is formed with characteristics significantly different from those of the bulk gel. In particular, in this layer, the porosity of agarose fiber network is significantly lower than in the bulk gel. The diffusion coefficient of solutes in this layer is consequently decreased for steric reasons. The diffusion characteristics are the crucial parameters reflecting the migration ability of diffusing particles which can be affected not only by the hydrogel structure but also their interactions with the active substance incorporated in the hydrogel. Chitosan, as the bio-functional material with high affinity to many harmful substances, was used in this study for the functionalizing of inert agarose hydrogel. The addition of chitosan as an active substance allowed to investigate the interactions directly in the motion of diffusing particles. Thus, the interactions can be included directly in the parameters obtained on the basis of diffusion experiments.

Studies dealing with the interactions of dyes with chitosan are often focused on traditional batch experiments (e.g., [21,22,23,24,25,26,27]). Some of them deal with hydrogels containing chitosan as an active substance in combination with other materials such as gelatin [28], pectin, DNA [29], activated carbon [30], Fe(III) [31], cellulose [32], and tri-polyphosphate [33]. Concepts for developing physical gels of chitosan and of chitosan derivatives are summarized in the review in [34]. As described in our previous study [9], the adsorption properties of chitosan are attributed to high hydrophilicity (due to OH groups), primary amino groups with high activity, and the flexible structure of polymer chains. This means that chitosan is a material with good affinity to different substances, including dyes, which are the subject matter of this study. Adsorbents based on chitosan have very good adsorption capacities and relatively low cost.

The studies on the transport of dyes and diffusion processes in chitosan materials are relatively scarce. Barron-Zambrano et al. [35] investigated the dynamic sorption of Reactive Black 5 onto chitosan in fixed-bed column. The obtained breakthrough curves were typical of systems that do not reach equilibrium which indicated that adsorption was affected by mass transfer limitations, probably due to intraparticle diffusion. It had a significant impact on column performance strongly affected by particle size. A smaller particle size resulted in a faster pore diffusion rate because the diffusion path was shorter and the resistance to diffusion was lower. Lazaridis and Keenan [36] used chitosan beads as barriers to the transport of azo dye in soil column. The used non-equilibrium transport models were divided into three parts: physical, chemical, and physical and chemical non-equilibrium transport. The application of a chitosan barrier resulted in a strong increase in the retardation factor of soil. García-Aparicio et al. [37] studied the diffusion of three small molecules, caffeine, theophylline and caprolactam, in chitosan gels with different concentrations of water by means of proton-localized NMR spectroscopy. The measured concentration profiles were in agreement with the Fickian law. The values of the diffusion coefficients ranged from 6.1 × 10^−10^ to 3.4 × 10^−10^ m^2^ s^−1^, depending on chitosan concentration and type of diffusant molecule. Cheung et al. [38] realized batch adsorption experiments with Orange 10, Acid Orange 12, Acid Red 18, Acid Red 73, and Acid Green 25. They concluded that the adsorption mechanism was predominantly intraparticle diffusion, but there was also a dependence on pore size as the dye diffuses through macropore, mesopore, and micropore, respectively. Similarly, two distinct linear parts were observed in plots of data obtained for the adsorption of Reactive Blue 4 dye onto Chitosan 10B. The initial linear portion may be attributed to the macropore diffusion and the second linear part to the micropore diffusion [21]. The intraparticle diffusion was presented as the rate-limiting process in the adsorption of dyes on double network gelatin/chitosan hydrogel [28], Reactive Black 5 on quartzite/chitosan composite [39], Rhodamine-6G on chitosan, nanoclay and chitosan–nanoclay composite [40], malachite green on chitosan beads [25,41], and indigo carmine on functional chitosan and β-cyclodextrin/chitosan beads [26]. Bilal et al. [42] developed Agarose-chitosan hydrogel-immobilized horseradish peroxidase and studied its bio-catalytic activity and effectivity in degradation of dye (Reactive Blue 19). Except for the main goals, the study provided detailed characteristics of hydrogel properties such as morphology and thermal stability.

Other studies are focused on the diffusion through chitosan membranes and films [15,43,44,45,46,47]. Hartig et al. [43] studied the diffusion of fructose in precipitated chitosan membranes using diffusion cells. They determined a diffusion coefficient which was dependent on the concentration and ranged between 6.2 × 10^−10^ and 2.1 × 10^−10^ m^2^ s^−1^. Yang et al. [44] performed permeation studies of model drug through preswollen chitosan/PVA blended hydrogel membranes using side-by-side diffusion cells. Similarly, Yang and Su [15] investigated the diffusion of 5-Fluorouracil through four kinds of chitosan membranes. They determined the permeability coefficient which was indirectly proportional to chitosan content. Waluga and Scholl [45] determined diffusion coefficients of different sugars and the sugar alcohol sorbitol in chitosan membranes and beads. Obtained diffusion coefficients ranged from 1.1 × 10^−10^ to 2.3 × 10^−10^ m^2^ s^−1^ for chitosan membranes, and from 1.4 × 10^−10^ to 2.4 × 10^−10^ m^2^ s^−1^ for chitosan beads. Xu et al. [46] incorporated four types of polyhedral oligosilsesquioxanes into chitosan by solution blending to fabricate composite membranes, and permeation studies were conducted for riboflavin. Their diffusion coefficients varied between 1.0 × 10^−12^ m^2^ s^−1^ and 2.7 × 10^−12^ m^2^ s^−1^. Carlough et al. [47] produced chitosan films and determined diffusion coefficients for Direct Red 81, Green 26, Blue 75, and Black 22. Their values (for 60 °C and pH 9) differed in magnitude and ranged from 4.5 × 10^−15^ m^2^ s^−1^ (Green 26) to 4.5 × 10^−14^ m^2^ s^−1^ (Black 22).

Our study is focused on the reactivity mapping of chitosan distributed in hydrogel during the dye migration. Since chitosan is considered a material with high potential to immobilize dyes, it is desirable to investigate the interactions in detail. In order to distinguish between the diffusivity through reactive and non-reactive medium, agarose hydrogel was chosen as the basic non-reactive material. Agarose hydrogel proved to be the suitable medium for the investigation of diffusion of different substances [10,11,47,48,49]. It can be enriched by an active substance which can interact with diffusing particles and (partially) immobilize them. Therefore, the interactions of diffusing dyes with active substance incorporated in the hydrogel can be studied in their motion. The main aim of this study is thus the detailed analysis of these two parallel processes.

## 2. Results and Discussion

**Figure 1:**
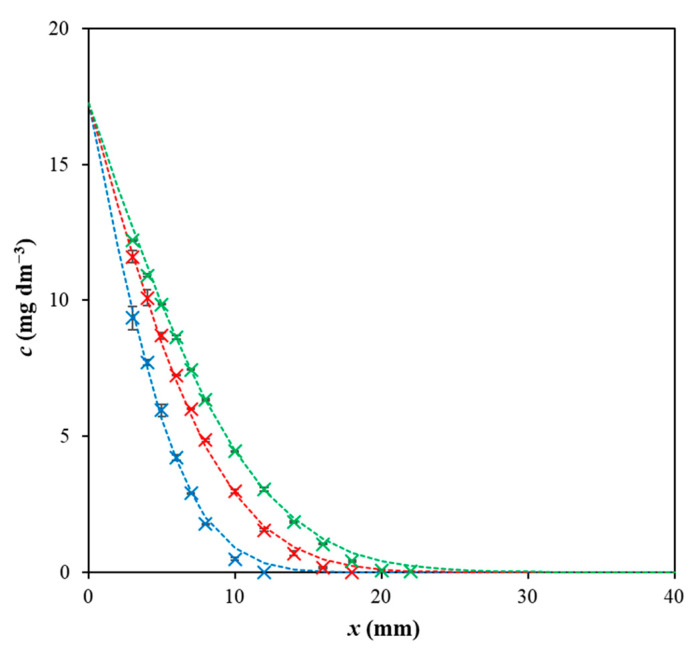
Concentration profiles of Direct blue 1 in agarose hydrogel after 24 h (blue), 48 h (red), and 72 h (green). Experimental data are fitted by Equation (1).

In Figure 1, the time development of a concentration profile in pure agarose hydrogel for Direct blue 1 is shown. Experimental data are fitted by Equation (1) derived on the basis of on Fick’s laws [9,50,51,52,53] and initial and boundary conditions listed in Table 1.

(1) $$c=c_{s}erfc\frac{x}{2\sqrt{D_{h}t}},$$

where *t* is time, *x* is distance from interface, *c* is concentration of dye, *c*_s_ is concentration at interface, and *D_h_* is the diffusion coefficient of dye in pure agarose hydrogel. If Equation (1) is applied for the data obtained for the diffusion of dyes in hydrogels enriched by chitosan, the diffusion coefficient *D_h_* in Equation (1) and (following) Equation (2) should be replaced by effective diffusion coefficient *D_ef_* (including interactions between dyes and chitosan). Both diffusion coefficients can be determined from the slope of the dependence of the total diffusion flux *m_t_* on the square root of time [9,50,51,52,53]:

(2) $$m_{t}=2c_{s}\sqrt{\frac{D_{h}t}{\pi }}$$

As can be seen, the concentration between the donor solution and hydrogel remained constant during the whole experiment as it agrees with the boundary condition (Table 1). Similarly, the hydrogel can be considered as the semi-infinite medium. This means that the hydrogel closer to the bottom of the cuvette remained free of dye during the whole diffusion experiment. The initial condition was that the initial concentration of dye in hydrogel was equal to zero. The conditions listed in Table 1 were valid for all realized experiments (for both types of hydrogels and all three dyes).

**Figure 2:**
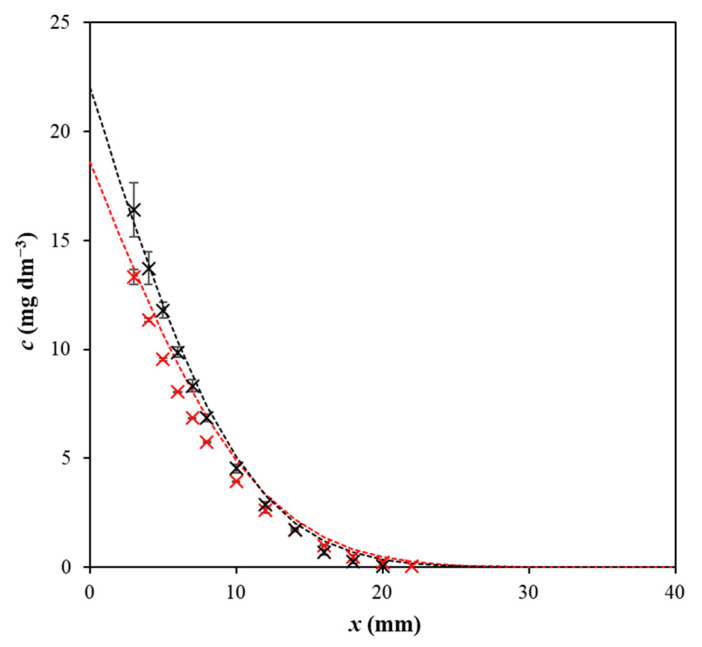
Concentration profiles of Direct blue 1 in agarose hydrogel (red), and hydrogel enriched by chitosan (black) after 72 h at pH 11. Experimental data are fitted by Equation (1).

The comparison of concentration profiles obtained for pure agarose hydrogel and the enriched one is shown in Figure 2. As can be seen, the profiles differ mainly in the distances close to the interface and the surface concentration *c_s_* is higher for the hydrogel enriched by chitosan. It was found that the surface concentrations are higher for hydrogel enriched by chitosan for all studied dyes and pH values.

**Figure 3:**
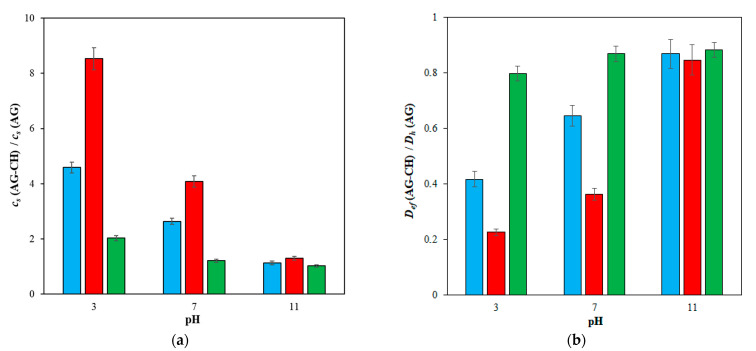
(**a**) The ratio between surface concentrations in hydrogels enriched by chitosan *c_s_* (AG-CH) and pure agarose hydrogels *c_s_* (AG); (**b**) the ratio between effective diffusion coefficient *D_ef_* (for hydrogel enriched by chitosan) and diffusion coefficients *D_h_* for pure agarose hydrogel; Direct blue 1 (blue), Sirius red F3B (red), and Reactive blue 49 (green).

The ratio between surface concentration in hydrogel enriched by chitosan and pure agarose hydrogel is strongly affected by character of dye and pH value (see Figure 3a). The ratios decreased with increasing pH values for all dyes, and they are not dependent on the duration of the experiment. It means that the surface concentration is constant for the given dye and given pH value. The ratios obtained for Reactive blue 49 at pH 7 and 11 are practically the same. The highest values were achieved for Sirius red F3B, the lowest ones for Reactive blue 49. However, the differences between dyes are negligible at pH 11. The results showed that the increase in diffusion rates and concentrations of dyes in the hydrogel enriched by chitosan (in comparison with pure agarose hydrogel) was caused mainly by the increase in surface concentration, which is a crucial factor determining the concentrations in hydrogels, as can be deduced from Equation (1). Another crucial factor is the (effective) diffusion coefficient which is lower in the hydrogel enriched by chitosan than in the pure agarose hydrogel (see Table 2 and Table 3). Therefore, the effect of the increase in the surface concentration preponderated over the effect of the decrease in the diffusivities of dyes.

The values of diffusion coefficients *D_h_* in Table 2 agree with values published for dyes in hydrogels [10,11,48,49,54,55,56,57,58,59]. Values of *D_ef_* obtained for hydrogels enriched by chitosan are lower as mentioned above (see Table 3 and Figure 3b). The highest diffusivity was determined for Reactive blue 49, the lowest for Direct blue 1. As can be seen, the diffusion coefficients *D_h_* changed with pH only slightly. They are practically independent from pH because errors of their determination are higher than the differences between values obtained for different pH values. The effective diffusion coefficients include the effect of interactions of dyes with chitosan. If we assume that the pore structure of hydrogel did not change with the addition of chitosan, the differences between *D_h_* and *D_ef_* should be caused mainly by the interactions. Rheological behaviour of agarose hydrogels and the hydrogels enriched by chitosan was investigated in detail in previous work [9]. Its results showed that the rheological behaviour of hydrogels was changed by the addition of chitosan. The changes were influenced by two contrary effects. The storage modulus was higher than the loss one and elastic character predominated for all studied hydrogels. However, the addition of chitosan caused the hydrogels to become more liquid and therefore, more permeable for diffusing particles. This effect can slightly suppress the decrease in the diffusivity of dyes in hydrogel containing chitosan. On the other hand, the decrease in *D_ef_* values (in comparison with *D_h_* ones) should be caused mainly by the dye–chitosan interactions.

While the ratio between surface concentrations in enriched and pure hydrogel decreased with increasing pH, the effect of pH on the mobility of dyes is the opposite. The common feature is that the diffusivities of studied dyes are comparable at pH 11. In neutral and acidic pH values, the decrease in diffusivity was stronger for Sirius red F3B and Reactive blue 49 and Direct blue 1 (in comparison with Reactive blue 49). The addition of chitosan into inert agarose hydrogel thus resulted in the increase in surface concentration and the decrease in the diffusion coefficient. Both parameters are mostly affected by the chitosan addition for Sirius red F3B. In this case, the biggest increase in surface concentration and the biggest decrease in diffusion coefficient were observed. The diffusivity of Sirius red F3B as well as its surface concentration are strongly influenced by pH value. In contrast, the effect of pH on the parameters determined for Reactive blue 49 was much weaker.

**Figure 4:**
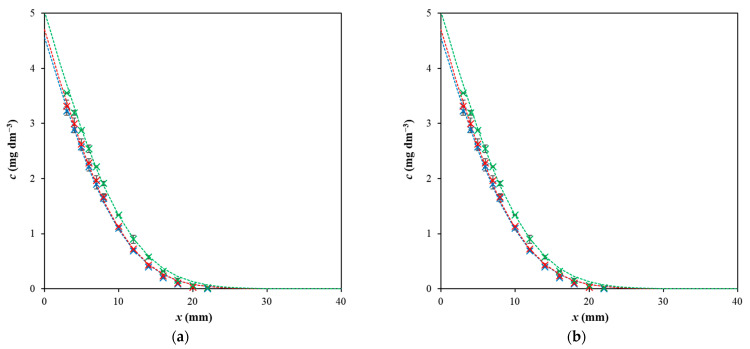
(**a**) The concentration profiles of Sirius red F3B in pure agarose hydrogel after 48 h; (**b**) the concentration profiles of Sirius red F3B in hydrogel enriched by chitosan after 48 h; pH 3 (blue), pH 7 (red), and pH 11 (green). Experimental data are fitted following Equation (1).

**Figure 5:**
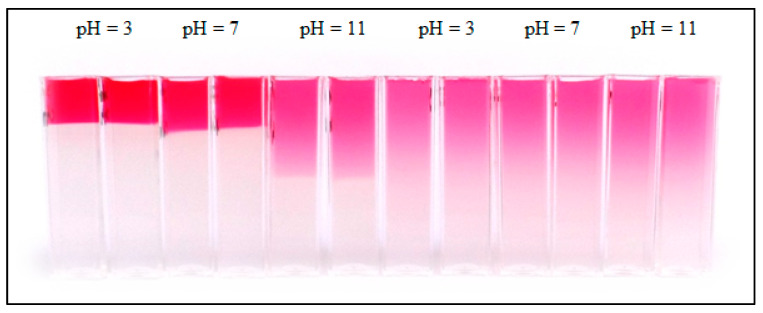
The concentration distribution of Sirius red F3B in hydrogel enriched by chitosan (**left**) and pure agarose hydrogel (**right**) after 72 h.

Both discussed parameters (surface concentration and diffusion coefficient) influenced the distribution of dyes in hydrogel in the diffusion. In Figure 4, the concentration profiles of Sirius red F3B in pure agarose hydrogel and hydrogel enriched by chitosan are compared. We can see that the profiles differed only slightly with pH changes when dyes were diffused in inert agarose hydrogel. In contrast, the changes in hydrogel containing chitosan as active substance are dramatical. The surface concentration at pH 3 is really high, which influenced the distribution of concentration in whole hydrogel. The difference between distribution of the dye in inert agarose hydrogel and hydrogel enriched by chitosan is shown in Figure 5. We can see that the dye particles diffuse in hydrogel containing chitosan as a layer with a sharp interface between hydrogel containing diffusing dye particles and hydrogel without them. We suppose that the reason for the formation of zones with a sharp boundary between coloured and transparent hydrogel are the electrostatic interactions between the amino group of chitosan and the sulfonic group of the dye. The amino groups are protonated at lower pH values [5,6,24,60], which resulted in their higher reactivity with dye and the formation of a concentration jump at a given distance from the interface between hydrogel and the donor dye solution. A similar sharp interface was observed for Direct blue 1.

**Figure 6:**
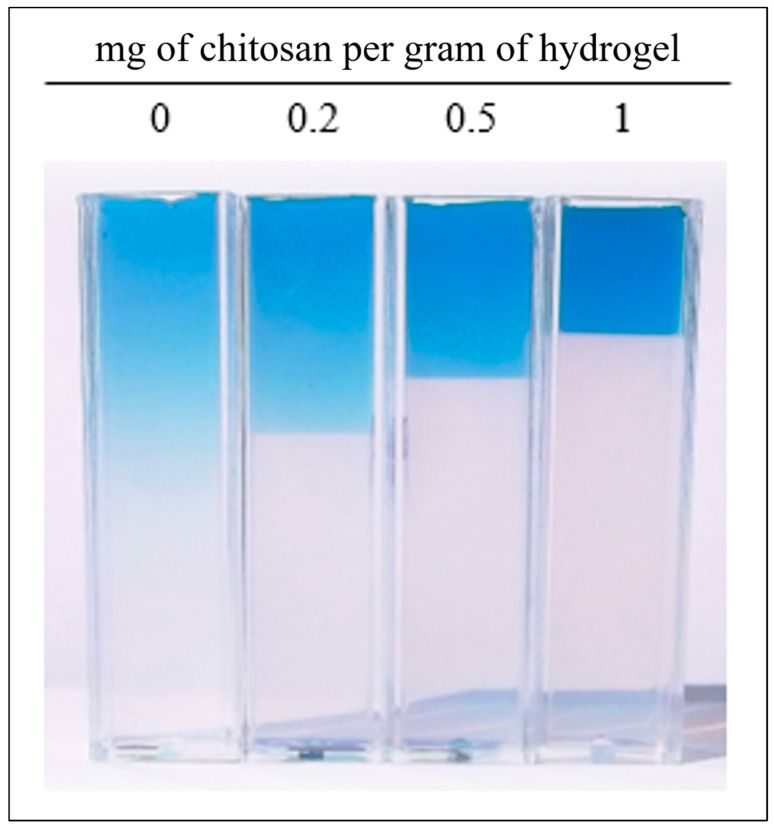
The concentration distribution of Direct blue 1 in hydrogels with different contents of chitosan after 72 h at pH 3.

On the basis of the obtained results, mainly having observed sharp interfaces between hydrogel containing dye and hydrogel without that, it was decided to realize additional diffusion experiments with Direct blue 1 and hydrogels with different contents of chitosan (similarly to previous work [9]). The obtained results are listed in Table 4. We can see that the diffusion coefficient gradually decreased with increasing content of chitosan. Simultaneously, the distribution of dye in hydrogel changed gradually into the sharply bordered dye layer as chitosan content gradually increased (see Figure 6). This effect is probably caused by electrostatic interactions between the amino group of chitosan and the sulfonic group of dyes. Since pure agarose hydrogel does not contain an active substance, the formation of zones with a sharp boundary between coloured and transparent hydrogel was not observed. The sharp interface was observed for all chitosan additions and this phenomenon was more pronounced for its larger amounts. Simultaneously, a deceleration of diffusion (related to a decrease in effective diffusion coefficient) was observed.

Since the effective diffusion coefficient is strongly affected by the interactions between dyes and chitosan as the active substance in the enriched hydrogel, we can analyse their relationship on the basis of the mathematical model published in previous work [9] and considering Fickian laws and a simple equilibrium between immobilized and free movable dye particles [50,51,52,53]. The relationship between the diffusion coefficient of dyes in pure agarose hydrogel (*D_h_*) and effective diffusion coefficient of dyes in hydrogels enriched by chitosan (*D_ef_*) can be expressed by the following equation [9,50,51,52,53]:

(3) $$D_{ef}=\frac{D_{h}}{\frac{c_{im}}{c_{free}}+1}=\frac{D_{h}}{K+1}=\frac{\mu D}{K+1}.$$

In Equation (3), the apparent equilibrium constant *K* represents the ratio between immobilized *c_im_* and free movable *c_free_* dye particles. It is supposed that the immobilization is caused by the interactions between dyes and chitosan. This simple equilibrium can be included into Fickian laws [9,50,51,52,53] and the value of *K* can be determined on the basis of Equation (4):

(4) $$K=\frac{D_{h}}{D_{ef}}-1.$$

The values of apparent equilibrium constants *K* are listed in Table 5. As can be seen, the values of *K* depended on the type of dye and pH value. In some cases, the values of *K* are greater than 1, which means that immobilized dye particles predominate over free mobile ones. In the opposite case (*K* < 1), most of the dye remains free and can migrate in hydrogel. Sirius red F3B can be strongly immobilized at acidic and neutral pH values, although the fraction of immobilized particles decreased, and free mobile particles predominated in alkaline environment. In contrast, free mobile particles predominated in the case of Reactive blue 49 and the decrease of *K* with increasing pH is only gentle. Direct blue 1 can be strongly immobilized in acidic conditions, but free mobile particles predominate in neutral and alkaline environment. Nevertheless, the local equilibrium between free movable and immobilized dyes cannot be considered as a stable state. It is dynamic, therefore, the values obtained here can be considered as average and effective. Another aspect is that it is assumed that chitosan particles are incorporated in hydrogel and trapped in their positions due to its non-diffusive dynamic state being strongly influenced by thermodynamic parameters (entropic traps) [61]. Chitosan, because of its structure, can fluctuate in its conformational arrangement, which can affect its reactivity.

**Figure 7:**
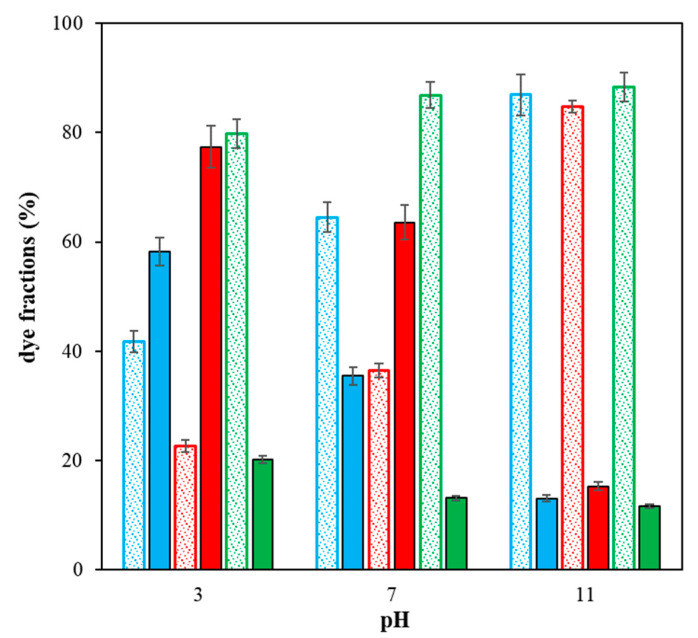
The portions of free mobile fraction (empty dotted columns) and immobilized dye fraction (full columns) in hydrogel enriched by chitosan; Direct blue 1 (blue), Sirius red F3B (red), and Reactive blue 49 (green).

Portions of free mobile fraction and immobilized dye fraction in hydrogel enriched by chitosan are shown in Figure 7. The calculation was based on the *K* values. We can see graphically the predomination of free mobile fraction for Reactive blue 49 as well as the strong immobilization in the case of Sirius red F3B (pH 3 and 7) and Direct blue 1 (pH 3).

Additional sorption experiments were conducted with both types of hydrogels in order to determine the efficiency of chitosan in agarose hydrogel for studied dyes. The results are listed in Table 6.

Experiments were conducted with aqueous solutions and no pH values were adjusted. Their aim was to compare adsorption efficiency of individual dyes (as such) without the presence of other substances and ions. As expected, the efficiency of enriched hydrogel was higher for Sirius red F3B and Direct blue 1 in comparison with Reactive blue 49. The results obtained for these dyes were comparable. Reactive blue 49 differed in the efficiency of pure agarose hydrogel as well as enriched one. The obtained values are in agreement with the results of diffusion experiments.

## 3. Conclusions

In this work, the transport properties of Direct blue 1, Sirius red F3B, and Reactive blue 49 in hydrogels were studied. Inert agarose hydrogel was enriched by chitosan as an active substance for the interactions with dyes. It was found that the presence of chitosan strongly affected the diffusion of dyes, mainly in the cases of Sirius red F3B and Reactive blue 49. Electrostatic interactions between the amino group of chitosan and the sulfonic group of dyes resulted in the formation of dye layers with a sharp interface between coloured hydrogel containing dye and transparent hydrogel without it. This effect was better observed in an acidic environment. The specific interaction between chitosan and dyes resulted in an increase in surface concentration and decrease in diffusivity. The decrease in diffusion coefficient caused by the interactions provided information about apparent equilibrium constant defined as the ratio between immobilized dye and free movable dye particles as well as their portions in enriched hydrogel. Immobilized particles predominated over dyes able to migrate in the cases of Sirius red F3B in acidic and neutral conditions and Direct blue 1 at pH 3.

The results obtained in this study provided information on reactivity mapping of dyes in hydrogel enriched by chitosan as an active substance. The advantage of this approach is the possibility to investigate the interactions of dyes with chitosan directly in their diffusion and characterize their transport affected by the interactions by means of a relatively simple mathematical model. The model is also usable for different bio-functional materials containing active sites for the immobilization of diffusing particles. Concentrations of free movable dyes were measured directly in hydrogels in defined distances from interfaces between hydrogel and donor solution. The experimental concentration profiles of dyes in hydrogels provided data for the determination of effective diffusion coefficients in which the effect of chemical interactions is included. Comparing with results obtained for inert agarose hydrogel, the fractions of free movable and immobilized particles can be calculated. This method is universal, its main requirements are the formation of hydrogel with defined size and shape, the possibility to determine a concentration profile in hydrogel, and the experimental arrangement corresponding with initial and boundary conditions given for the mathematical model.

## 4. Materials and Methods

### 4.1. Chemicals

Chitosan (medium molecular weight), agarose (routine use class), and Direct blue 1 were purchased from Sigma Aldrich (St. Luis, MO, USA). Sirius red F3B and Reactive blue 49 were purchased from Synthesia (Pardubice, Czech Republic). Acetic acid for the preparation of chitosan solution was purchased from Lachner (Neratovice, Czech Republic). Disodium hydrogen phosphate, sodium dihydrogen phosphate, citric acid, and sodium hydroxide for the preparation of buffer solutions were purchased from Penta (Chrudim, Czech Republic).

The exact molecular weights of chitosan and agarose were determined by means of size exclusion chromatography coupled with multiangle static light scattering, differential refractive index, and UV/VIS detection (SEC chromatographic system from Agilent Technologies, detectors from Wyatt Technology). The exact molecular weights were 251 ± 4 kDa for chitosan and 146 ± 3 kDa for agarose.

The deacetylation degree of chitosan was determined by potentiometric titration as described by Garcia et al. [62]. The degree was determined as 83.8 ± 0.2% mol.

### 4.2. Preparation of Hydrogels

The preparation of hydrogels was based on the thermo-reversible gelation of agarose solution described in previous works [9,50,51,52,53]. Agarose hydrogel gelatinized from the solution of agarose in water. The agarose content in hydrogel was 10 mg g^−1^. The mixture was slowly heated with continuous stirring up to 80 °C, stirred at this temperature in order to obtain a transparent solution, and finally sonicated (1 min) to remove gasses. Afterwards, the mixture was slowly poured into the PMMA spectrophotometric cuvette (inner dimensions: 10 × 10 × 42 mm). The cuvette orifice was immediately covered with a pre-heated plate of glass to prevent drying and shrinking of gel. The flat surface of the boundary of resulting hydrogels was provided by wiping an excess solution away. Gentle cooling of cuvettes at the laboratory temperature led to the gradual gelation of the mixture.

Agarose–chitosan hydrogels were prepared from agarose solution mixed with the solution of chitosan. An accurately weighed amount of chitosan was dissolved in 50 cm^3^ of acetic acid (5% wt.) The solution was titrated by 1M NaOH up to pH equal to 7 and diluted by distilled water (the final volume was 100 cm^3^). The agarose content in hydrogel was 10 mg g^−1^, the content of chitosan was 1 mg g^−1^.

### 4.3. Diffusion Experiments

Two cuvettes (with both types of hydrogels) were placed into 250 cm^3^ of dye solution. Dye solutions were prepared in buffers with pH equal to 3, 7, and 10. Buffers were composed of disodium hydrogen phosphate, sodium dihydrogen phosphate, citric acid, and sodium hydroxide in appropriate ratios. The bulk concentration of dyes was 50 mg dm^−3^. The solution was stirred continuously by the magnetic stirrer and the dye were left to diffuse from the solution into the hydrogels through the square orifices of the cuvettes. Diffusion experiments were triplicated, it means that three different vessels for the same type of dye were used. The durations of the diffusion experiments were 24, 48 and 72 h. In these time intervals, the cuvettes were taken out of the solution and the UV-VIS spectra were measured in dependence on distances from the interface between hydrogel and donor solution. Varian Cary 50 UV–VIS spectrophotometer (Agilent Technologies, Palo Alto, CA, USA) equipped with the special accessory providing controlled fine vertical movement of the cuvette in the spectrophotometer was used for this purpose [21,28,40]. The concentration of dyes was determined at different positions in the hydrogels by means of a calibration line. The spectra were calibrated for the hydrogels with the known concentration, homogeneously distributed in the whole volume of the hydrogel.

The experiments were performed at laboratory temperature (25 ± 1 °C). Data are presented as average values with standard deviation bars.

### 4.4. Sorption Experiments

Glass tubes (length and diameter, 1 cm) were filled by hydrogels and placed separately into vessels with 20 cm^3^ of dye solution. Vessels were closed and covered by parafilm to prevent evaporation. Diffusion experiments were triplicated, which means that three different vessels for the same type of dye and the same type of hydrogel were used. Hydrogels were taken out after 6 days and the UV-VIS spectra of solutions were collected. The decrease in concentration and the sorption efficiency were determined on the basis of calibration line.

Experiments were performed at laboratory temperature (25 ± 1 °C). Data are presented as average values with standard deviation bars.

## Figures and Tables

**Table 1:** Initial and boundary conditions of diffusion experiments.

Time *t*	Distance *x*	Concentration *c*
*t* = 0	*x* > 0	*c* = 0
*t* > 0	*x* = 0	*c* = *c_s_*
*t* > 0	*x* → ∞	*c* = 0

**Table 2:** Values of diffusion coefficient (*D_h_*) determined for pure agarose hydrogel.

Dye	*D_h_* (pH 3) (m^2^ s^−1^)	*D_h_* (pH 7) (m^2^ s^−1^)	*D_h_* (pH 11) (m^2^ s^−1^)
Direct blue 1	(1.51 ± 0.05) × 10^−10^	(1.55 ± 0.07) × 10^−10^	(1.54 ± 0.10) × 10^−10^
Sirius red F3B	(2.05 ± 0.12) × 10^−10^	(2.04 ± 0.10) × 10^−10^	(2.03 ± 0.12) × 10^−10^
Reactive blue 49	(2.98 ± 0.09) × 10^−10^	(2.90 ± 0.13) × 10^−10^	(3.02 ± 0.07) × 10^−10^

**Table 3:** Values of effective diffusion coefficient (*D_ef_*) determined for hydrogel enriched by chitosan.

Dye	*D_ef_* (pH 3) (m^2^ s^−1^)	*D_ef_* (pH 7) (m^2^ s^−1^)	*D_ef_* (pH 11) (m^2^ s^−1^)
Direct blue 1	(6.32 ± 0.37) × 10^−11^	(1.00 ± 0.05) × 10^−10^	(1.34 ± 0.11) × 10^−10^
Sirius red F3B	(4.64 ± 0.22) × 10^−11^	(7.42 ± 0.37) × 10^−11^	(1.72 ± 0.13) × 10^−10^
Reactive blue 49	(2.36 ± 0.13) × 10^−10^	(2.52 ± 0.05) × 10^−10^	(2.67 ± 0.08) × 10^−10^

**Table 4:** Values of diffusion coefficient (*D_h_*) and effective diffusion coefficient (*D_ef_*) determined for Direct blue 1 and pH 3 for different contents of chitosan.

*D_h_* (m^2^ s^−1^)	*D_ef_* (m^2^ s^−1^)		
Without Chitosan	0.2 mg g^−1^	0.5 mg g^−1^	1 mg g^−1^
(1.51 ± 0.05) × 10^−10^	(1.32 ± 0.03) × 10^−10^	(9.83 ± 0.16) × 10^−11^	(6.32 ± 0.37) × 10^−11^

**Table 5:** Values of effective diffusion coefficient (*D_ef_*) determined for hydrogel enriched by chitosan.

Dye	*K* (pH 3)	*K* (pH 7)	*K* (pH 11)
Direct blue 1	1.39 ± 0.08	0.55 ± 0.03	0.15 ± 0.01
Sirius red F3B	3.42 ± 0.23	1.75 ± 0.12	0.18 ± 0.01
Reactive blue 49	0.25 ± 0.01	0.15 ± 0.01	0.13 ± 0.01

**Table 6:** Adsorption efficiency of pure agarose hydrogels and hydrogels enriched by chitosan.

Dye	Pure Agarose Hydrogels	Enriched Hydrogels
Direct blue 1	(0.84 ± 0.02)%	(21.26 ± 0.61)%
Sirius red F3B	(1.14 ± 0.03)%	(24.48 ± 0.44)%
Reactive blue 49	(3.37 ± 0.06)%	(12.70 ± 0.17)%

## Data Availability

Data will be available on request.
